# Assessment of Cardiovascular Risk and Examination of Blood Klotho Levels in Patients with Ankylosing Spondylitis

**DOI:** 10.3390/jcm15010131

**Published:** 2025-12-24

**Authors:** Burcu Dogan, Aysel Tocoglu, Sabah Tuzun, Ulku Akcay, Ayfer Altas, Emel Gonullu, Ali Tamer

**Affiliations:** 1 Department of Family Medicine, Gulhane Faculty of Medicine, University of Health Sciences, Ankara 06010, Türkiye; 2 Division of General Internal Medicine, Department of Internal Medicine, Sakarya University Training and Research Hospital, Sakarya 54100, Türkiye; agurkan@sakarya.edu.tr (A.T.); drulkuyilmaz@gmail.com (U.A.); ayferaltas@sakarya.edu.tr (A.A.); tamer@sakarya.edu.tr (A.T.); 3 Department of Family Medicine, Haseki Training and Research Hospital, Istanbul 34096, Türkiye; sabahtuzun@gmail.com; 4 Division of Rheumatology, Department of Internal Medicine, Sakarya University Training and Research Hospital, Sakarya 54100, Türkiye; emelgonullu@sakarya.edu.tr

**Keywords:** ankilozan spondilartrit, cardiovascular diseases, Klotho, plasma atherogenic index, SCORE

## Abstract

**Background/Objectives:** Ankylosing Spondylitis (AS) is associated with increased cardiovascular disease risk due to chronic systemic inflammation. The Atherogenic Index of Plasma (AIP) and Systematic Coronary Risk Evaluation (SCORE) are valuable tools for cardiovascular risk assessment, while Klotho, an anti-aging protein with cardioprotective properties, may serve as a potential biomarker for cardiovascular health. Recent studies have shown that soluble α-Klotho contributes to vascular protection by increasing endothelial cell proliferation, reducing apoptosis, and enhancing angiogenic capacity, thereby helping to maintain microvascular integrity. We aimed to assess cardiovascular event risk in AS patients using AIP and SCORE and investigate the relationship between serum Klotho levels and these factors. **Methods:** A case–control study was conducted between August and September 2019. The study included 24 AS patients and 24 healthy controls aged 18 and above, with 13 females and 11 males. **Results:** No significant difference was found in serum Klotho levels between the AS and control groups in terms of SCORE and AI classifications. In the high-risk SCORE classification group, AI was found to be elevated at 0.42. In the AS group, Klotho levels were observed as 0.73 in the low-risk group, 0.60 in the moderate-risk group, and 0.61 in the high-risk group (*p* = 0.974). When evaluating HDL levels, Klotho was determined to be 7.29 ± 6.81 for HDL < 35 and 0.60 [0.33] for HDL ≥ 35 (*p* = 0.036). **Conclusions:** An AI exceeding 0.40 in the high-risk SCORE group and in patients with active disease according to the BASDAI score indicated an increased cardiovascular event risk in the AS group. Further studies are warranted regarding serum Klotho levels, HDL, and LDL subclasses in AS patients.

## 1. Introduction

Ankylosing spondylitis (AS) is an autoimmune chronic inflammatory disease primarily affecting the sacroiliac and vertebral column joints, categorized under seronegative spondyloarthropathy (SPA). While peripheral joint involvement is less common, extra-articular manifestations can also occur, potentially affecting renal, ophthalmic, cardiac, pulmonary, or neurological systems [1,2,3,4,5]. Similar to other chronic systemic inflammatory conditions like rheumatoid arthritis and psoriatic arthritis, AS has been associated with cardiac and vascular pathologies. The heightened risk of cardiovascular complications due to AS severity and disease duration contributes to a higher mortality rate compared to the general population. The pronounced systemic inflammation seen in AS has been linked to an elevated risk of cardiovascular disease [6].

The European League Against Rheumatism (EULAR) and the Assessment of Spondyloarthritis International Society (ASAS) state that there is an increased risk of cardiovascular disease (CVD) in patients with inflammatory joint disorders (IJD) compared to the general population. This underscores the significance of rheumatic diseases like AS on cardiovascular health and emphasizes the need to consider CVD risk factors in the management of these diseases. The EULAR task force has proposed the use of the Systematic Coronary Risk Evaluation (SCORE) system, particularly for special populations such as those with rheumatic diseases, to more accurately assess cardiovascular risk [7].

In recent times, numerous algorithms have emerged to aid in assessing cardiovascular disease (CVD) risk in asymptomatic individuals. Notably, the SCORE (Systematic Coronary Risk Evaluation) algorithm stands out as a significant tool used to predict the likelihood of experiencing cardiovascular events over the next decade. Based on data from 12 prospective studies conducted across 11 European countries, the SCORE system tracks the health of over 205,000 individuals from the general population, equivalent to approximately 2.1 million person-years [8]. Specifically designed to calculate the risk of fatal cardiovascular events, the SCORE system takes into account various factors, including age, gender, blood pressure, cholesterol levels, and smoking status [9]. By integrating these parameters, the SCORE system serves as a valuable tool for estimating an individual’s risk of cardiovascular events. Such predictive tools play a crucial role in assisting healthcare professionals in evaluating patients’ cardiovascular risk profiles and implementing appropriate preventive measures.

The plasma atherogenic index (AIP) is considered a predictive marker for rapid plaque progression (RPP) and is evaluated independently of traditional cardiovascular risk factors. AIP is defined as the logarithm of the ratio of plasma triglyceride (TG) concentrations to high-density lipoprotein cholesterol (HDL), based on 10, i.e., log (TG/HDL-C) [10]. In individuals with rheumatic diseases, particularly for managing the risk of atherosclerotic cardiovascular disease (ACD), the European League Against Rheumatism (EULAR) strongly recommends the use of the classical atherogenic index (CAI) [7].

The Klotho gene is predominantly expressed in the distal tubule cells of the kidney, parathyroid glands, and choroid plexus of the brain. This gene encodes a single-pass transmembrane protein [11,12]. Three different types have been identified: α-Klotho, beta-Klotho, and γ-Klotho. In terms of genetic predisposition to cardiovascular diseases, the polymorphism of the Klotho gene has been associated [12,13,14]. α-Klotho binds to FGF receptors on renal tubular cell membranes to bind FGF23. It has been characterized as a systemic anti-aging hormone and is known to inhibit lipid peroxidation and inflammation. Positive effects on endothelial dysfunction, oxidative stress, and cellular antioxidant defense have been demonstrated [13,15,16]. Recent studies have shown that soluble α-Klotho contributes to vascular protection by increasing endothelial cell proliferation, reducing apoptosis, and enhancing angiogenic capacity, thereby helping to maintain microvascular integrity [15]. In cases of Klotho deficiency, conditions such as atherosclerosis, hypertension, neovascularization disorders, cardiac fibrosis, sinoatrial node dysfunction, cardiac electrical activity disturbances, coronary heart disease, and inflammatory diseases may arise [11,13,17,18,19]. Hypertension and atherosclerosis patients have been found to exhibit a 45% decrease in circulating α-Klotho levels [20]. Reactivation of endogenous Klotho production or exogenous Klotho supplementation may alleviate renal and cardiac fibrosis, delay the progression of chronic kidney disease, improve mineral metabolism, enhance cardiomyopathy, and reduce vascular calcification [13,21,22]. Additionally, a decrease in cardiovascular and non-cardiovascular mortality has been observed in patients maintaining high serum Klotho levels [23]. The positive effects of recombinant Klotho administration in patients with uremic cardiomyopathy have highlighted the use of recombinant Klotho for prophylactic and therapeutic purposes as a consideration. Recently, studies were conducted on the clinical application of recombinant Klotho for the prevention and treatment of atherosclerosis [24].

Our study on the evaluation of cardiovascular risk using plasma AIP and SCORE classification in AS patients is crucial for understanding the cardiovascular health status of these patients and implementing appropriate measures. If a significant relationship is found between plasma Klotho levels and AIP as well as SCORE classification, it may indicate the potential of Klotho in modulating cardiovascular risk factors. This could pave the way for the development of new approaches in the diagnosis and treatment of patients.

This study differs from previous work by examining blood Klotho levels together with the Atherosclerosis Index (AI) and SCORE-based cardiovascular risk in patients with Ankylosing Spondylitis (AS). Its relevance comes from emphasizing the importance of broadening cardiovascular risk assessment in AS beyond conventional lipid measures, indicating that biomarkers such as Klotho, which, together with LDL and HDL subclasses, may provide a more detailed understanding of subclinical atherosclerosis in this patient group.

## 2. Materials and Methods

The study was conducted as a cross-sectional study between 1 August 2019 and 1 September 2019. Patient files were scanned retrospectively. It included 24 patients aged 18 and above diagnosed with Ankylosing Spondylitis (AS) according to the New York criteria [25], who were followed up in the rheumatology, internal medicine, and family medicine clinics of Sakarya University Training and Research Hospital. Individuals who visited the Family Medicine outpatient clinic for periodic health examinations and had no known diseases were included in the healthy group. Exclusion criteria for the study were defined as follows: smoking, alcohol consumption, malignancy, diabetes, hypertension, heart disease, chronic use of medications (antihypertensive agents, antidiabetic agents, antihyperlipidemic agents, antiepileptic agents, antidepressants, antithyroid agents, anti-Parkinson drugs), hypertriglyceridemia (TG > 400 mg/dL), acute or chronic infection, acute or chronic kidney failure, chronic obstructive pulmonary disease, and body mass index (BMI) above 30 kg/m^2^.

Case and control groups were matched 1:1 based on age and gender, comprising 24 healthy, randomly selected volunteers. The same exclusion criteria used for selecting AS patients were also applied to the control group.

In our study, hemogram and C-reactive protein (CRP) levels were evaluated as inflammation parameters. In addition, the evaluation of cardiovascular risk utilized the SCORE classification and AIP. The SCORE scoring system is a tool used to calculate cardiovascular risk, taking into account the individual’s age, gender, systolic blood pressure, smoking status, LDL cholesterol, and HDL cholesterol levels. Cardiovascular risk is categorized into four classes: low, moderate, high, and very high. Since our participants were under 40 years old, the updated SCORE 2 classification could not be utilized. The AIP is calculated as the logarithm of the ratio of plasma triglycerides to HDL cholesterol levels. This index serves as an indicator in assessing cardiovascular risk. Typically, AIP values between −0.3 and 0.1 are associated with low risk, values between 0.1 and 0.24 are associated with moderate risk, and values above 0.24 are associated with high cardiovascular risk.

Both patient and control groups provided fasting blood samples in the morning to determine α-Klotho levels after an overnight fast. Plasma samples were collected and immediately centrifuged at 3000 rpm for 15 min. The samples were then stored at −80 °C in Eppendorf tubes (Eppendorf AG, Hamburg, Germany) until α-Klotho level measurements were conducted, which occurred approximately within one month of collection.

The determination of Klotho levels was performed using the Klotho ELISA kit (Shanghai Yehua Biological Technology Co., Ltd., Shanghai, China). A volume of 40 μL from each sample was utilized for analysis using the microELISA method. Test results were evaluated using a Multiskan™ GO Microplate Spectrophotometer (Thermo Scientific™, Waltham, MA, USA) at a wavelength of 450 nm. Data acquisition and analysis were performed using Multiskan™ GO software (version 1.01, Thermo Scientific™, Waltham, MA, USA). The sensitivity of the test was 0.01 ng/mL within the range of 0.5–20 ng/mL.

### Statistical Analysis

The data were analyzed using SPSS version 29. Descriptive statistical methods, including frequency, percentage, mean, standard deviation, median, and interquartile range, were employed. Additionally, Student’s *t*-test was used to evaluate continuous variables exhibiting a normal distribution, while the Mann–Whitney U test and Kruskal–Wallis test were utilized for assessing variables displaying abnormal distribution. The Chi-square test was employed for examining categorical data. A significance level of *p* < 0.05 was considered statistically significant for all data analyses. Due to the insufficient sample size and the limited distribution of events in this study, it was not feasible to perform subgroup analyses with adequate methodological reliability. Therefore, the statistical evaluations presented are restricted to descriptive analyses rather than adjusted models.

## 3. Results

Our study included a total of 48 participants. The case group comprised 24 (50.0%) individuals diagnosed with AS, while the control group consisted of 24 (50.0%) healthy individuals. Table 1 presents the cardiovascular risk factors between the case and control groups. Table 1 includes the age, gender, systolic blood pressure, smoking status, LDL cholesterol levels, HDL cholesterol levels, and other important clinical characteristics of both groups.

Table 2 summarizes the SCORE classification and atherogenic index of the case and control groups, including the cardiovascular risk classification and atherogenic index values of both groups. No statistically significant differences were detected between the atherogenic index of the AS patient group (AI 0.40 [0.24]) and the control group (AI 0.31 [0.31]) (*p*: 0.375). Additionally, no significant difference was observed in SCORE risk assessments between AS patients and the control group (*p*: 0.403).

The median Klotho level of the participants in the case group was determined to be 0.61 [0.25] ng/mL, while the median Klotho level of the control group was measured as 0.62 [3.73] ng/mL. No statistically significant difference was observed between the groups (*p* = 0.279).

No significant relationship was found between Klotho and hemogram parameters or CRP in AS patients (*p* > 0.05 for all). In the healthy group, a relationship was observed between Klotho and platelets; however, no relationship was found between other hemogram parameters and CRP (r = 0.482, *p* = 0.017 and *p* > 0.05, respectively).

When Klotho levels were evaluated among the SCORE classification groups, in the control group, they were determined as 0.64 [8.43] in the low-risk group, 0.66 [4.58] in the moderate-risk group, and 0.60 [7.48] in the high-risk group (*p* = 0.889). In the case group, they were observed as 0.73 [0.66] in the low-risk group, 0.60 [0.19] in the moderate-risk group, and 0.61 [0.08] in the high-risk group (*p* = 0.974).

When evaluated among the SCORE classification groups, no significant difference was found in the PAI in the case group (*p* = 0.645). However, when the PAI was assessed according to the SCORE classification, in the control group, it was determined as 0.11 [0.47] in the low-risk group, 0.16 [0.31] in the moderate-risk group, and 0.42 [0.09] in the high-risk group (*p* = 0.012).

In both case and control groups, no significant relationship was found between Klotho level and AI groups categorized as 0.24 and above, and below 0.24 (*p* = 0.742 and *p* = 0.151, respectively).

In various studies, it has been reported that there is an increase in cardiovascular risk (CVR) in individuals with a specific threshold value (0.23) above the AI classification. Dobiásová’s study identified high CVR in groups where AI was 0.40, associated with conditions such as diabetes, hypertension, hyperlipidemia, and male gender. A comparison of Klotho levels between groups with high and low AI was conducted. When the AI cutoff value was assessed at 0.40, no significant difference in Klotho levels was detected between high and low AI groups in AS or healthy groups (*p* = 0.881 for the AS group and *p* = 0.887 for the healthy group). In addition, when the AI cutoff value was assessed at 0.24, no significant difference in Klotho levels was detected between high and low AI groups in both groups (*p* = 0.820 for the AS group and *p* = 0.209 for the healthy group). Table 3 displays the Klotho levels in groups with high and low AI.

The relationship between LDL/HDL ratio and Klotho levels was found to be not significant in both healthy (*p* = 0.424) and AS (*p* = 0.322) groups. When HDL was evaluated for increased cardiovascular risk (CVR), Klotho levels were determined as follows: Klotho for HDL < 35: 7.29 ± 6.81, Klotho for HDL ≥ 35: 0.60 [0.33] (*p* = 0.036). Klotho levels tended to be higher than Klotho levels in the low HDL group.

For AS patients, 2 (16.7%) had low CV, 4 (33.3%) had moderate CV, 3 (25.0%) had high, and 3 (25.0%) had very high cardiovascular (CV) risk according to the BASDAI score (*p* = 0.315). Among patients with inactive disease according to the BASDAI score, the AI was 0.29 ± 0.21, while among those with active disease, the AI was 0.42 [0.39] (*p* = 0.410). In patients with inactive disease according to the BASDAI score, Klotho was 0.61 [0.34], while in those with active disease, Klotho was 0.61 [0.23] (*p* = 0.843).

## 4. Discussion

Although there are many studies on the increased risk of cardiovascular disease in AS patients, there have been few studies examining the relationship between Klotho levels and parameters such as SCORE and AI (Atherosclerotic Index) in AS patients. Therefore, it may be difficult to compare our findings with other studies. Factors such as smoking, diabetes, hypertension, hyperlipidemia, along with medication use and gender, are well-known to have an impact on cardiovascular risk [26]. In our study group, which was matched for age and gender, we evaluated these factors as exclusion criteria in order to control their effects. This approach can enhance the accuracy of the results and help focus on the content of the research.

In previous studies, it has been found that AI values in male AS patients are significantly higher compared to healthy controls. In the study conducted by Cure et al., AI was found to be significantly higher in male AS patients with a value of 0.23 compared to healthy controls [27]. In Dobiásová’s study, values reaching 0.4 were observed in men and in patients with cardiovascular risk factors such as diabetes, hypertension, and dyslipidemia [28]. In our study, the mean AI value in AS patients was found to be 0.40, indicating high values. This supports the consideration of AS-diagnosed patients as having high cardiovascular risk similar to those with chronic diseases such as diabetes, hypertension, and hyperlipidemia. The uniqueness of our study lies in including both male and female AS patients, allowing for the evaluation of the effect of gender on AI values. Larger sample sizes and long-term follow-up studies may help to further validate these findings. In our study, no significant difference was observed in terms of AI according to the SCORE classification groups. While AI values in the control group increased from low-risk to high-risk SCORE classification groups, this increase was not significant. However, AI was found to be high at 0.42 in the high-risk group according to the SCORE classification. This emphasizes the importance of using SCORE classification and AI values together in assessing cardiovascular risk. Such studies are believed to contribute to better patient management and improvement of cardiovascular health in clinical practice.

Our study found no significant difference in serum Klotho levels between the AS and control groups. Additionally, there was no significant difference in Klotho levels between the SCORE and AI groups. In both the control and AS groups, no statistically significant difference in Klotho levels was observed among low, moderate, and high SCORE risk groups. However, among AS patients classified as low risk by SCORE, Klotho levels were higher compared to other groups. This suggests a potential relationship between Klotho levels and AS, as well as high cardiovascular risk. Further research is needed to clarify this relationship and evaluate causality.

The relationship between serum Klotho levels and changes, and the development of cardiovascular damage and increased morbidity and mortality, is well known [13,29]. In our study, no significant difference was found in Klotho levels between the AS and control groups. Additionally, no significant difference in Klotho levels was detected between the SCORE and AI groups. In both the control and AS groups, there was no statistically significant difference in Klotho levels among low, moderate, and high SCORE risk groups (Table 4). However, among AS patients classified as low risk by SCORE, Klotho levels were higher (0.73 [0.66]) compared to other groups. This may suggest a potential relationship between Klotho levels and not only AS but also high cardiovascular risk. Due to insufficient studies in this area, further research is needed to assess the clarity of this relationship and its cause-and-effect nature (Table 5).

It was observed that LDL and HDL levels in the AS group were lower compared to the control group, but this difference was not statistically significant. The HDL levels associated with coronary events mentioned in the PROCAM study are emphasized to be below 35 mg/dL. The average HDL level in the AS group was 45.5 mg/dL, while in the control group, it was 53.0 mg/dL. These values were found not to be below the risky HDL value indicated in the PROCAM study. Arking et al. noted that the dysfunctional variant of the Klotho gene is inversely associated with HDL-C levels [30]. Jiang et al. found a complex relationship between serum Klotho levels and plasma HDL-C and LDL-C levels, represented by dose–response curves in a “U” or inverted “U” shape. Our study also supported this by showing a significantly higher Klotho level at 6.96 for HDL < 35 [31]. As demonstrated in the study by Kanda et al., it has been interpreted that soluble α-Klotho levels in serum may be associated with different HDL subclasses [32]. It is believed that a more comprehensive evaluation can be achieved by examining different levels of HDL subclasses in relation to blood Klotho levels and other risk factors.

The association of increased cardiovascular disease risk with AS may be attributed to the prominent systemic inflammation characteristic of this condition [33]. Disease activity is typically assessed using the Bath Ankylosing Spondylitis Disease Activity Index (BASDAI), with patients having higher BASDAI scores generally considered to have active disease [34]. In our study, patients with BASDAI scores of 4 or higher were identified as having active disease. The values of SCORE, API, and Klotho levels in these patients were compared with those of patients with BASDAI scores lower than 4. The analysis did not reveal a significant difference in Klotho levels between patients with BASDAI scores of 4 or higher and those with lower scores. It was considered that the limited number of patients with AS in our study may have influenced this result.

The study excluded patients with non-AS chronic diseases and adopted broad exclusion criteria, which could enhance the clarity and focus of the results. However, this led to a low number of AS patients participating in the study. Given the limited sample size and restricted event distribution, reliable subgroup analyses could not be performed. Accordingly, the statistical results presented are descriptive in nature and should be interpreted within these methodological constraints. The limitations of the study were reported as the limited number of participants and the inability to examine LDL and HDL subclasses. The failure to evaluate cardiac parameters such as NT-proBNP, CK-MB, and echocardiography is another limitation of the study.

## 5. Conclusions

The study indicates that the Ankylosing Spondylitis (AS) group demonstrates an increased cardiovascular risk in terms of the Atherosclerosis Index (AI). No significant difference in blood Klotho levels was found between the AS and control groups in terms of SCORE and AI classifications. Although not statistically significant, Klotho levels were found to be lower in individuals with a high SCORE risk in both the AS and healthy groups (0.60; 0.61, respectively). This suggests the need for additional studies to evaluate the impact of blood Klotho levels on cardiovascular risk in AS patients. Assessing LDL and HDL subclasses alongside Klotho levels may better help determine cardiovascular risks in AS patients with the aid of biomarkers.

## Figures and Tables

**Table 1:** The cardiovascular risk factors between the ankylosing spondylitis and healthy groups.

	Ankylosing Spondylitis Group (n = 24)	Healthy Group (n = 24)	*p*
Age (years)	45.1 ± 10.8	44.9 ± 10.5	0.936 *
Gender			1.000 **
Female	13 (%54.2)	13 (%54.2)	
Male	11 (%45.8)	11 (%4.58)	
LDL-cholesterol	131.0 [52.0]	148.0 [74.0]	0.509 ***
HDL-cholesterol	46.8 ± 10.1	56.3 ± 15.9	0.018 *
LDL/HDL ratio	3.01 [1.24]	2.78 ± 1.19	0.529 ***
Hemoglobin	12.5 [2.5]	13.46 ± 1.54	0.027 ***
Leukocyte	7357.50 ± 1981.01	6414.58 ± 1768.52	0.089 *
Neutrophil	4340.0 ± 1464.56	3450.0 [1740.0]	0.044 ***
Lymphocyte	2264.04 ± 947.44	2043.33 ± 498.63	0.318 *
Platelet	284,000.0 [43,250.0]	237,250.0 ± 55,160.04	0.006 ***
CRP	3.40 [10.98]	0.0 [4.16]	0.042 ***
Systolic blood pressure	110.0 [30.0]	105.0 [20.0]	0.122 ***
Diastolic blood pressure	70.0 [18.0]	70.0 [14.0]	0.275 ***
Waist circumference	98.0 [16.0]	99.3 ± 10.8	0.893 ***
BMI	27.8 ± 5.4	26.0 ± 4.1	0.213 *
Fat percentage	30.7 ± 10.1	28.8 ± 7.6	0.470 *
Abdominal fat	38.8 ± 10.0	36.7 ± 7.6	0.450 *
BASDAI			-
<4 points	12 (%50.0)	-	
≥4 points	12 (%50.0)	-	

* Student *t* test, ** Ki-kare test, *** Mann–Whitney U test.

**Table 2:** The SCORE classification and atherogenic index of the case and healthy groups.

	Ankylosing Spondylitis Group (n = 24)	Healthy Group (n = 24)	*p*
Scores groups			0.403 *
Low risk	4 (%16.6)	8 (%33.3)	
Medium risk	10 (%41.7)	7 (%29.2)	
High risk	7 (%29.2)	8 (%33.3)	
Very high risk	3 (%12.5)	1 (%4.2)	
Plasma Atherogenicity Index	0.40 [0.24]	0.31 [0.31]	0.375 **
Plasma Atherogenicity Index (≥0.40)			0.242 *
Low	12 (%50.0)	16 (%66.7)	
High	12 (%50.0)	8 (%33.3)	
Plasma Atherogenicity Index (≥0.24)			0.233 *
Low	7 (%29.2)	11 (%45.8)	
High	17 (%70.8)	13 (%54.2)	

* Ki-kare test, ** Mann–Whitney U test.

**Table 3:** Klotho level according to different plasma atherogenicity index cut-off values in the healthy group and AS group.

	Klotho Level		
	Ankylosing Spondylitis Group (n = 24)	Healthy Group (n = 24)	*p*
Plasma Atherogenicity Index (≥0.40)			
Low (n:28 (%58.3))	0.60 [3.73]	0.60 [0.33]	0.423 *
High (n:20 (%41.7))	0.64 [7.47]	0.61 [0.22]	0.270 *
Plasma Atherogenicity Index (≥0.24)			
Low (n:18 (%37.5))	0.68 [4.59]	0.59 [0.09]	0.151 *
High (n:30 (%62.5))	0.62 [5.09]	0.61 [0.51]	0.742 *
Scores groups			
Low risk	0.64 [8.43]	0.73 [0.66]	0.933
Medium risk	0.66 [4.58]	0.60 [0.19]	0.230
High risk	0.60 [7.48]	0.61 [0.08]	0.955
Very high risk	-	-	-

* Mann–Whitney U test.

**Table 4:** The treatment categories according to systematic coronary risk evaluation risk groups in patients with ankylosing spondylitis.

Treatment Category		Ankylosing Spondylitis (n = 24)	Systematic Coronary Risk Evaluation Risk Groups				*p*
			Low	Moderate	High	Very High	
Use of NSAID (Ibuprofen, Naproxen, Diclofenac)	No	7 (%29.2)	1 (%25.0)	6 (%60.0)	0 (%0.0)	0 (%0.0)	0.033
	Yes	17 (%70.8)	3 (%75.0)	4 (%40.0)	7 (%100.0)	3 (%100.0)	
Use of Glucocorticoid	No	21 (%87.5)	4 (%100.0)	9 (%90.0)	6 (%85.7)	2 (%66.7)	0.606
	Yes	3 (%12.5)	0 (%0.0)	1 (10.0)	1 (%14.3)	1 (%33.3)	
Use of Conventional synthetic DMARDs (csDMARDs)	No	17 (%70.8)	3 (%75.0)	7 (%70.0)	6 (%85.7)	1 (%33.3)	0.419
	Yes	7 (%29.2)	1 (%25.0)	3 (%30.0)	1 (%14.3)	2 (%66.7)	
Use of Biologic DMARDs (bDMARDs)	No	9 (%37.5)	2 (%50.0)	4 (%40.0)	2 (%28.6)	1 (%33.3)	0.907
	Yes	15 (%62.5)	2 (%50.0)	6 (%60.0)	5 (%71.4)	2 (%66.7)	
Use of Targeted synthetic DMARDs (tsDMARDs)	No	23 (%95.8)	4 (%100.0)	9 (%90.0)	7 (%100.0)	3 (%100.0)	0.691
	Yes	1 (%4.2)	0 (%0.0)	1 (%10.0)	0 (%0.0)	0 (%0.0)	

Ki-kare test.

**Table 5:** Treatment category-based Klotho and plasma atherogenicity index levels in ankylosing spondylitis patients.

Treatment Category		Ankylosing Spondylitis Group (n = 24)	Klotho	*p*	API	*p*
Use of NSAID (Ibuprofen, Naproxen, Diclofenac)	No	7 (%29.2)	0.64 [0.35]	0.288	0.34 [0.25]	1.000
	Yes	17 (%70.8)	0.59 [0.20]		0.40 [0.28]	
Use of Glucocorticoid	No	21 (%87.5)	0.60 [0.21]	0.620	0.40 [0.20]	0.505
	Yes	3 (%12.5)	0.64 [-]		0.03 [-]	
Use of Conventional synthetic DMARDs (csDMARDs)	No	17 (%70.8)	0.61 [0.34]	0.494	0.40 [0.21]	0.576
	Yes	7 (%29.2)	0.59 [0.07]		0.39 [0.58]	
Use of Biologic DMARDs (bDMARDs)	No	9 (%37.5)	0.65 [0.30]	0.096	0.40 [0.46]	0.770
	Yes	15 (%62.5)	0.59 [0.07]		0.39 [0.22]	
Use of Targeted synthetic DMARDs (tsDMARDs)	No	23 (%95.8)	0.60 [0.29]	-	0.40 [0.24]	-
	Yes	1 (%4.2)	-		-	

Mann–Whitney U test.

## Data Availability

The data that support the findings of this study are available from the corresponding author upon reasonable request.
